# Supplementary material for: Accelerated Long-Fragment Circular PCR for Genetic Manipulation of Plant Viruses in Unveiling Functional Genomics
Source: Viruses. 2023 Nov 28;15(12):2332. doi: 10.3390/v15122332 (PMC10747169; doi:10.3390/v15122332)
Supplement: Supplementary file 1 [file viruses-15-02332-s001.zip › viruses-2665397-supplementary.pdf]

Supplementary Figures and Tables

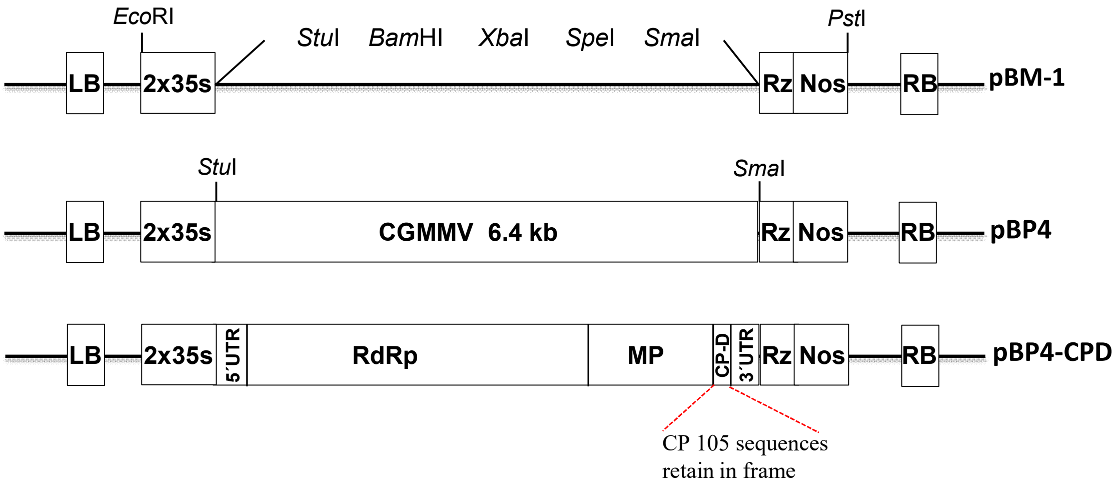

Supplementary Figure S1: Schematic diagram of modified binary vector, CGMMV infectious clone, and partial CP deletion constructs were developed for the current studies. pBM: The vector presence of the left border of T-DNA (LB), a double 35S promoter (2x35S), multiple cloning sites (MCS), a ribozyme sequence (Rz), a NOS terminator (NOS), and a right border of T-DNA (RB). pBP4: The vector contains a full-length CGMMV clone derived from pBMCG6.4(Ref:32). pBP4-ΔCP: The 105 bp of 5' end of CP gene sequences were retained and after sequences have been deleted up to 3' end of CP gene. (RdRp: RNA-dependent RNA polymerase, MP: Movement protein, CP: Coat protein, UTR: Untranslated region, Black line: indicates restriction enzymes as well as MCS, Red Line: indicates CP retain part).

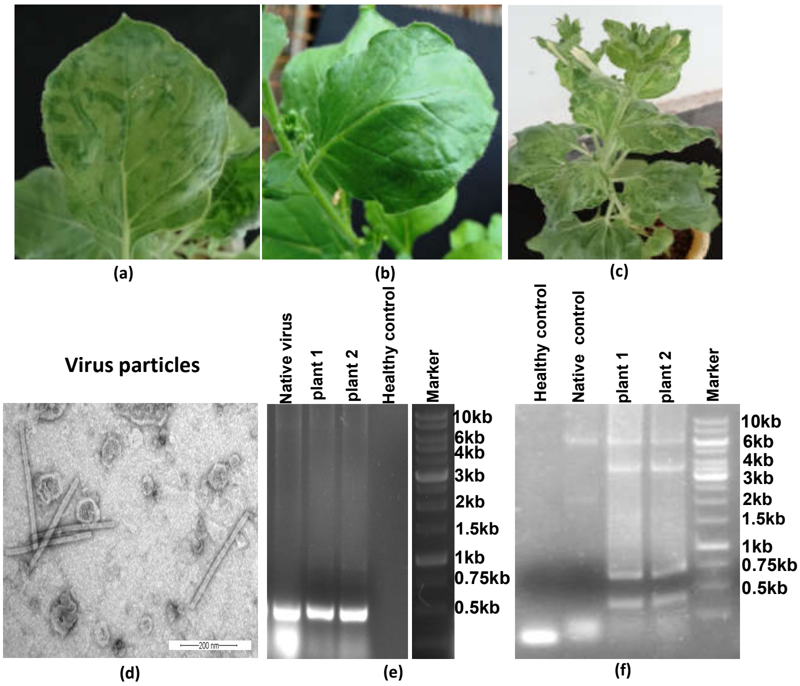

Supplementary Figure S2: Infectivity analysis of the full-length CGMMV clone. (a) The mottle mosaic symptoms showing in *Nicotiana benthamiana* systematic leaf with inoculation of pBMCG6.4 infectious clone, (b) Health leaf of *N. benthamiana*, (c) CGMMV purified virus inoculated leaf was showing mottle mosaic symptoms,(d) Electron micrograph showing rod-shaped particles of CGMMV in *N. benthamiana* plant inoculated with pBP4 agro construct. Further, the infection of CGMMV pBP4 was confirmed from the inoculated symptomatic leave samples through RT-PCR using primers specific for (e) CP gene, and (f) full-genome of CGMMV.

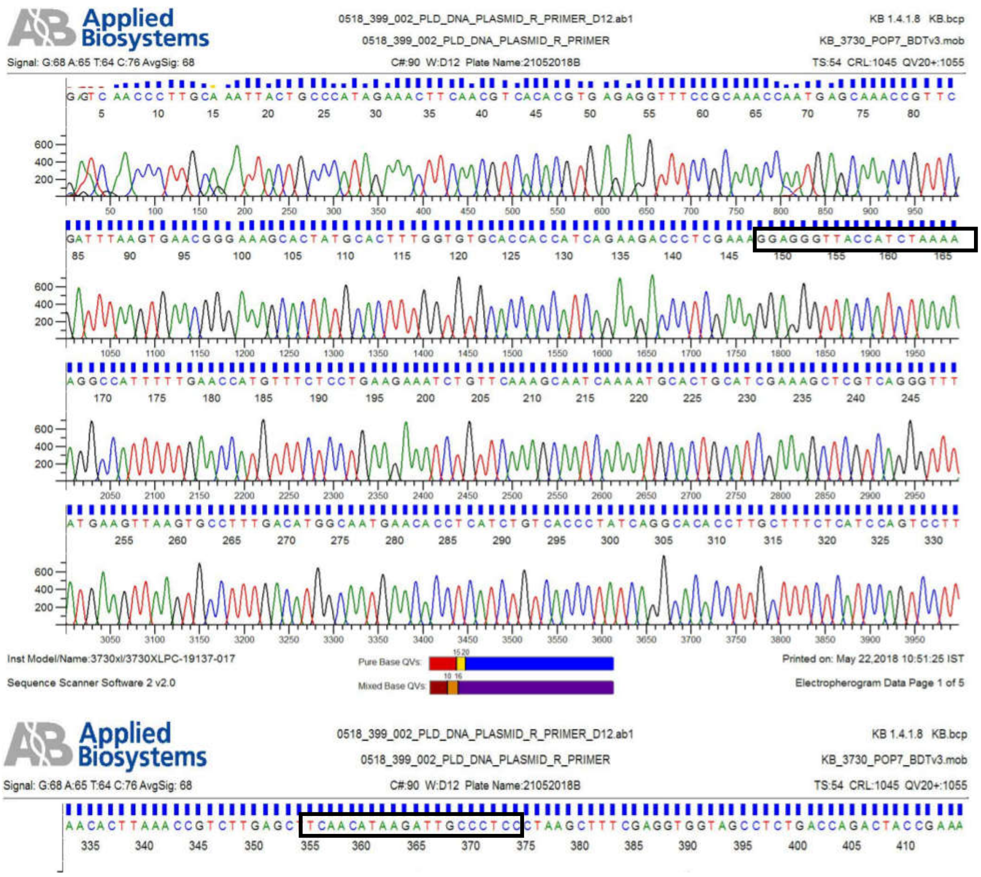

Supplementary Figure S3: DNA sequence based confirmation of the insertion of *NbPDS* gene into CGMMV genome-based gene silencing vector (VIGS) by LC-PCR. The dark black boxes indicate the location of PDS gene insertion in between the coat protein andUTR of CGMMV. The sequencing results show the gene insertion created at the desired location with our choice of place and without any modification, and additionally, proved that LC-PCR did not add an extra nucleotide in the CGMMV genome during the gene insertion procedure.

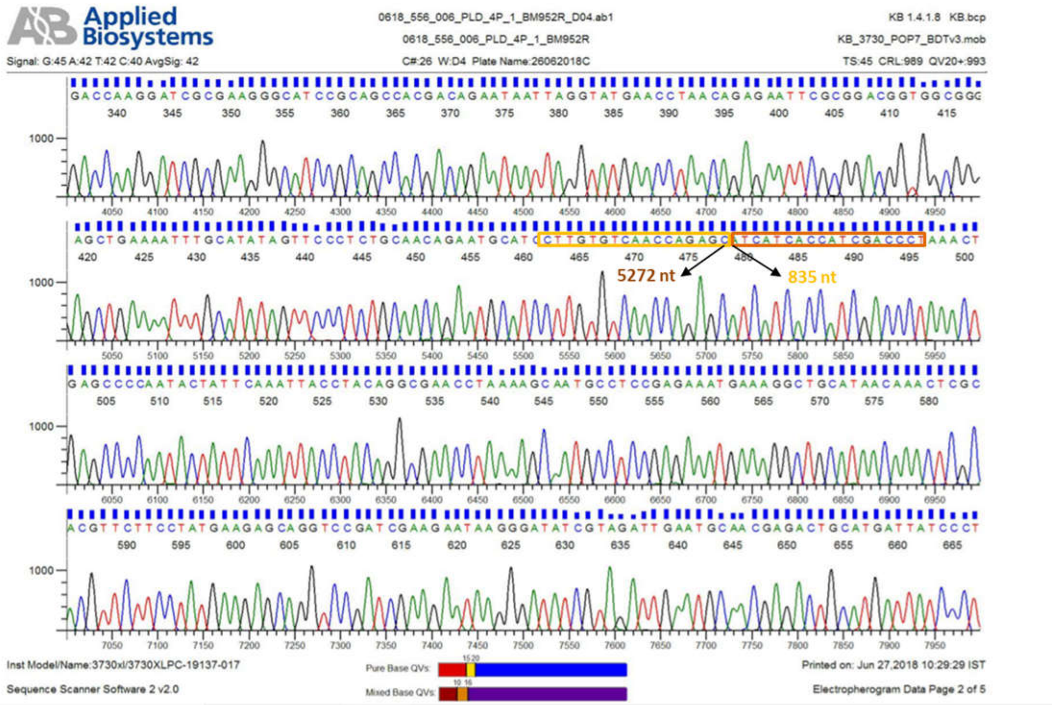

Supplementary Figure S4: The DNA sequence-based confirmation of partial deletion of RNA-dependent RNA polymerase (RdRP) and movement protein (MP) genes from the CGMMV genome by LC-PCR. The red box indicates the start position (835nt) of RdRp deletion and the yellow box indicates the end position (5272 nt) of MP deletion. The black arrow shows the deleted genomic part.

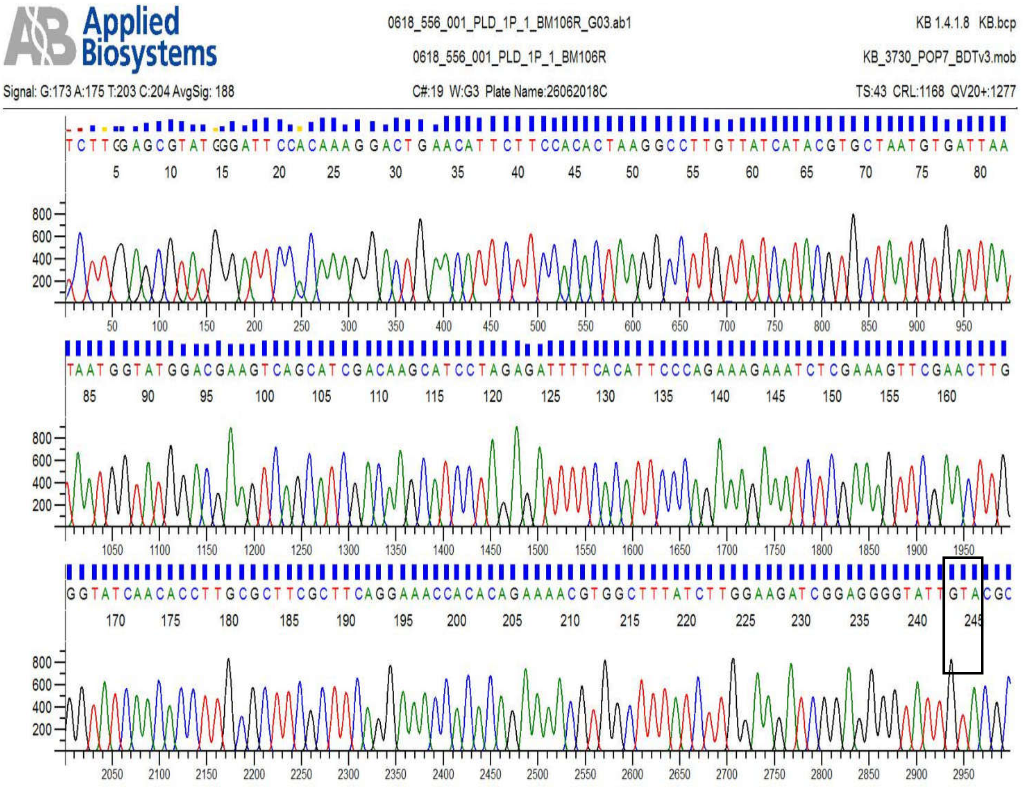

Supplementary Figure S5: The DNA sequence confirms the single amino acid substitution (silent mutation) in the CGMMV genome by LC-PCR. The vertical black box showing the exact mutation created in the RdRP gene of CGMMV genome.

Supplementary Table S1: Primers used for infectious clone development and presence of confirmation CP, NbPDS, ToLCNDV-CP, housekeeping gene of ACT1 in plant samples.

Primer Name	Primer sequence (5'- 3')	Location	Nucleotide coordinates	Expected amplicon size(kb)	Purpose
BM 486F	***CGGGATCC***GTTTTAATTTTTAAAATTAAAC	5´UTR	1-22	6.44	Binary vector construction
BM 489R	***GCTCTAGA***TGGGCCCTTACCCAGG	3´UTR	6426-6442		
BM 556F	***CGGGATCC***ATGGCTTACAATCCGATC	CP	5763-5781	0.48	CGMMV CP detection
BM 557R	***CGGAATTC***AGCTTTCGAGGTGGTAGC	CP	6230-6248		
BM 566R	***GCTCTAGAAAGCTT***AAGTCCTGACGGGAACATAAGAAG	CP	5808-5832		Deletion construction
BM 849F	GGTATTGTGTTGGACTCGGG	ACT1	219-238	0.16	Semi Quantitative PCR
BM 859R	GCTGTGGTAGTGGATGAGTAAC	ACT1	361-382		
BM1013F	CCTCACGCCCAACTAAAC	Nb PDS	534-551	0.18	Semi Quantitative PCR
BM1014R	CAACCCAGTCTCGTACCAAT	Nb PDS	695-715		
BM909F	GGAGGGCAATCTTATGTTGAAGC	Nb PDS	968-990	0.23	RT-PCR detection
BM910R	AGGAGGGTTACCATCTAAAAAGG	Nb PDS	1173-1196		
BM1017F	***GGATCC***ATGGCGAAGCGACCAGCAGA	ToLCNDV-CP		0.77	RT-PCR detection
BM1018R	***TCTAGA***GTGGTGGTGGTGGTGGTGTTAATTTGTTACCGAATCAT	ToLCNDV-CP			
